# Chromosome-level genome assembly of *Chouioia cunea* Yang, the parasitic wasp of the fall webworm

**DOI:** 10.1038/s41597-023-02388-5

**Published:** 2023-07-26

**Authors:** Ziqi Wang, Xingzhou Ma, Jiachen Zhu, Boying Zheng, Ruizhong Yuan, Zhaohe Lu, Xiaohan Shu, Yu Fang, Shiji Tian, Qiuyu Qu, Xiqian Ye, Pu Tang, Xuexin Chen

**Affiliations:** 1 State Key Lab of Rice Biology, Zhejiang University, Hangzhou, 310058 China; 2 Zhejiang Provincial Key Laboratory of Biology of Crop Pathogens and Insects, Zhejiang University, Hangzhou, 310058 China; 3 Ministry of Agriculture Key Lab of Molecular Biology of Crop Pathogens and Insects, Zhejiang University, Hangzhou, 310058 China; 4 Institute of Insect Sciences, College of Agriculture and Biotechnology, Zhejiang University, Hangzhou, 310058 China

**Keywords:** Entomology, Genome

## Abstract

*Chouioia cunea* Yang 1989 is a parasitic wasp of many lepidopteran insects during their pupal stage, and has been successfully used to control pests such as the fall webworm *Hyphantria cunea*. Here we reported the chromosome-level genome of *C. cunea* by using short (MGI-SEQ), long (Oxford Nanopore), chromatin-linked (Hi-C) sequencing reads and transcriptomic data, representing the first chromosome-level genome of parasitic wasps of the family Eulophidae. The total assembly length is 171.99 Mb, containing 6 pesudo-chromosomes with a GC content of 36.89% and the scaffold/contig N50 length of 31.70/26.52 Mb. The BUSCO completeness of the assembly was estimated to be 98.7%. A total of 12,258 protein-coding genes (PCGs), 10,547 3′-UTRs, and 10,671 5′-UTRs were annotated. This high-quality genome is an important step toward a better understanding of the genomes of the Eulophidae (Chalcidoidea), and will serve as a valuable resource for analyses of phylogenetic relationships and the evolution of Hymenoptera.

## Background & Summary

*Chouioia cunea* Yang is a species of small, gregarious endoparasitoid wasp of the family Eulophidae belonging to Chalcidoidea (Hymenoptera)^1^. This species efficiently parasitizes a wide range of lepidopteran insects. It is native to East Asia and has been introduced to various regions of the world, including North America, where it has been used as a potential biological control agent. In China, *C. cunea* has been successfully used to suppress populations of the fall webworm, *Hyphantria cunea* Drury (Lepidoptera: Arctiidae), an invasive pest in East Asia. The fall webworm has been reported to feed on more than 600 plant species, including apple, boxelder, cherry, mulberry, plum, sycamore and walnut^2–4^. It is well known principally for its larval stage, which creates the characteristic webbed nests on the tree limbs in the late summer and fall. These webs allow for the finding of mates, temperature regulation, increased growth rate, and protection from predators^4^, making *H. cunea* one of the most destructive insect pests. The biological control programs for the fall webworm in China have been facilitated by the development of mass rearing technology for *C. cunea*. The biology of this parasitoid is well studied, including the parasitism rate, the number of offspring produced per parasitized host, as well as host preferences^5,6^. However, less was known about the molecular mechanisms by which *C. cunea* controls host populations.

**Fig. 1:**
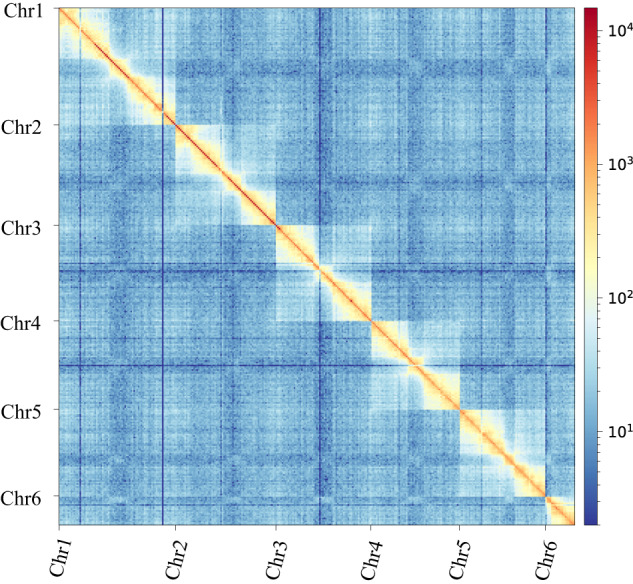
The genome features of *C. cunea*: genome-wide Hi-C heatmap of chromatin interaction counts. The scale bar represents the interaction frequency of Hi-C links.

**Fig. 2:**
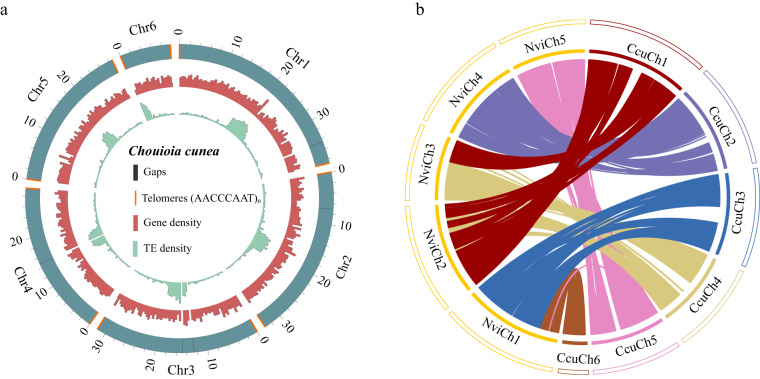
(**a**) Circos plot of the 6 chromosomes of *C. cunea*. The scale bar represents Mbp. Blue: Pseudo-chromosomes length in Mb unit; Red: Gene density; Green: TE density; Orange: Telomeres; Black: Gaps. (The size of Orange highlight does not represent the length of telomeres.) (**b**) Synteny between *C. cunea* and *N. vitripennis*. Coloured arcs indicate homologous genomic blocks between species.

A high-quality reference genome is essential for advancing genetics and genomic research. However, to date, no genome of Eulophidae has been reported, and only a limited number of chromosomal–level assemblies are available for Chalcidoidea. In this study, we applied MGI-SEQ short-read, ONT long-read and Hi-C sequencing technologies to obtain a chromosome-level genome assembly for *C. cunea*, which represents the first chromosome-level genome assembly of the Eulophidae. The genome assembly size of *C. cunea* was 171.99 Mb with the scaffold/contig N50 length being 31.70/26.52 Mb (Table 2). The assembly of Hi-C technology scaffolding reached chromosome-level with six pseudo-chromosomes containing only five gaps (Fig. 1, 2). 26.06 Mb repeat sequences were identified, accounting for 15.15% of the *C. cunea* genome. A total of 12,258 protein-coding genes were predicted. Additionally, phylogenetic analyses based on single-copy genes were constructed to understand the relationship between *C. cunea* and other species. As the first high-quality chromosome-level genome assembly for Eulophidae, this genome is a valuable resource for enhancing our understanding of the evolutionary relationship of chalcidoids and enabling comparative studies on the biology, behavior, and genetic evolution of *C. cunea*.

## Methods

### Insect collection and sequencing

The species *Chouioia cunea* were originally obtained from the Keyun company in Henan Province, China. All insect colonies were maintained in an environment-controlled room (26 ± 1°C, 60 ± 10% RH and 16: 8 h L: D photoperiod). The voucher specimens were stored in 100% ethanol and kept in the Parasitic Hymenoptera Collection of the Institute of Insect Sciences, Zhejiang University. DNA samples were extracted from adult individuals using Blood & Cell Culture DNA Mini Kit (Qiagen, Hilden, Germany). Long-reads and short-reads (library insert size: 350 bp) sequencing were performed on Nanopore PromethION platform (Oxford Nanopore Technologies, UK) and MGISEQ. 2000 (MGI, Shenzhen, China), respectively. In total, 48.88 Gb long reads (~284.2 coverage) and 25.37 Gb short reads (~147.5 coverage) were generated (Table 1). Short-reads transcriptomes of larvae and adults were sequenced on the same platforms as DNA samples. For full-length transcriptome, the Invitrogen Trizol kit was used to extract RNA. Purified mRNA isolated from adult individuals were fragmented with divalent cations under increased temperature. These short fragments were used as templates to synthesize the first-strand cDNA using hexamer primers and superscriptTMIII (InvitrogenTM, Carlsbad, CA, USA). Second-strand cDNA was then synthesized in a solution containing buffer, dNTP, RNaseH, and DNA polymerase I and subsequently purified using a QiaQuick PCR extraction kit (Qiagen). EB buffer was used to resolve these short fragments for end reparation and poly A addition. The sequence adaptors were linked to two ends of short cDNA sequences, and suitably sized cDNA fragments were selected out for PCR amplification based on the agarose gel electrophoresis results. Finally, the library established was sequenced using a PromethION platform. The total data generated from full-length sequencing was 13.06 Gb, while the total data generated from short-read sequencing was 20.99 Gb (Table 1).

**Table 1:** Statistics of the DNA/RNA sequence data used for genome assembly.

Library	Insert size (bp)	Reads number	Raw data (Gb)	N50 Read Length (bp)	Sequence coverage (X)
MGI	350	169,141,850	25.37	150	147.5
ONT	20,000	7,168,006	48.88	19,385	284.2
Hi-C	350	172,862,644	25.93	150	150.8
RNA-seq (MGI)	350	139,960,058	20.99	150	—
RNA-seq (ONT)	—	11,760,877	13.06	1,532	—
Total	—	500,83,435	134.23	—	—

The Hi-C library was constructed using the restriction endonuclease DpnII. In brief, the adult individuals of *C. cunea* were fixed with formaldehyde and crosslinking was stopped by adding glycine. The fixed tissue was then grounded to powder before re-suspending in nuclei isolation buffer to obtain a suspension of nuclei. The purified nuclei were digested with DpnII and marked by incubating with biotin-14-dATP. Biotin-14-dATP from non-ligated DNA ends was removed owing to the exonuclease activity of T4 DNA polymerase. The ligated DNA was sheared into 300−600 bp fragments, and then was blunt-end repaired and A-tailed, followed by purification through biotin-streptavidin-mediated pull down. Finally, a total of 25.93 Gb Hi-C data (~150.8 coverage) was generated after sequencing on an MGISEQ. 2000 platform (Table 1).

### ***De novo*** genome assembly

The raw reads were cleaned and filtered by fastp v0.19.4^7^ with default parameters. The genome size of *C. cunea* was estimated using GenomeScope v1.0.0^8^ and the 17-mer distribution was analyzed via Jellyfish v2.3.0^9^. Based on the total number of 5,232,854,730 17-mers and a peak 17-mer depth of 33, the genome size of *C. cunea* was estimated to be 158.57 Mb, and the estimated heterozygosity rate was approximately 0.80%.

We first downloaded the mitochondrial genome of *Sclerodermus guani* from NCBI (GenBank: MH748670.1) as a reference. The ONT reads were aligned to the mitochondrial genome reference using Minimap2 v2.1^10^. The aligned reads were self-corrected using Canu v2.1.1^11^. The corresponding mitochondrial reads were manually assembled into a circular mitochondrion and polished using Nextpolish v1.3.1^12^ by short reads. To assemble the nuclear genome of *C. cunea*, NextDenovo v2.3.1 (https://github.com/Nextomics/NextDenovo) was used for genome assembly with the default parameters. Because mitochondria can cause over-polishing to the nuclear mitochondrial (NUMT) region, it is important to include the mitochondrial genome before genome polishing^13,14^. The mitochondrial sequence was tandemly replicated twice and added as a single contig to the end of the assembly. We performed two rounds of long reads correction using Racon v1.4.28^15^ and one round of short reads correction using Nextpolish. The mitochondrial contig was discarded after the polish was finished. Purge_dups v1.2.5^16^ was used to remove haplotypic duplication. Next, 3D de novo assembly (3D-DNA)^17^ was used to assemble each draft into a candidate chromosome-length assembly. Juicebox v1.22^18^ viewed the HiC-map of each assembly. The completeness of the assemblies was evaluated by the BUSCO v4.1.4^19^ (in the insecta_Odb10 database^20^) to be 98.70%, including 1,323 single-copy genes and 26 duplicated genes (Table S2). For gap-filling and telomere discovery, the error-prone long reads were corrected first by Fmlrc2^21^ with short reads and then self-corrected using Canu. The corrected ONT reads > 50 KB were selected. The non-repeat region of surrounding sequences of both sides of the gap was used as a query to search the raw draft assembly and the corrected long reads using blastn and MUMmer v3.5^22^. The ends of super scaffolds were queried to the corrected reads to find reads containing telomeric repeat. The Hi-C heatmap of our final assemblies was plotted and visualized by hicPlotMatrix v3.6^23^. The final version of the genome assembly of *C. cunea* was highly contiguous with a total assembly length of 171.99 Mb contained in 11 contigs. The scaffold/contig N50 length was 31.70/26.52 Mb (Table 2). Meanwhile, the assembly of Hi-C scaffolding reached chromosome-level with 6 pesudo-chromosomes, and there are only 5 gaps in chromosome scaffolds by manual curation and gap filling, indicating that the assembled genome in this study was of high quality (Figs. 1, 2).

**Table 2:** Genome assembly and annotation statistics of *Chouioia cunea*.

Elements		Current Version
Genome assembly	Assembly size (Mb)	171.99
	Number of scaffolds/contigs	6/11
	Longest scaffold/contig (Mb)	38.83/34.64
	N50 scaffold/contig length (Mb)	31.70/26.52
	GC (%)	36.89
	Number of Gaps	5
	BUSCO completeness (%)	98.70
Gene annotation	Protein-coding genes	12,258
	BUSCO completeness (%)	98.70
	5′ UTRs	10,671
	3′ prime UTRs	10,547
	mRNAs	16,982

### Repeat annotation

The filtered library was combined by RepBase and Dfam v3.2^24^ referring to Insecta. This data set was then used as a library to search the TEs on *C. cunea* using RepeatMasker v4.1.2^25^. Species-specific repeat library was generated using RepeatModeler v2.1.0 (http://www.repeatmasker.org/RepeatModeler/) with default settings and these identified repeat elements were classified using a reference-based similarity search against a species-specific repeat library in RepeatMasker. Next, the annotation results of repeat elements were merged against the filtered library and species-specific repeat library. To estimate sequencing depth, ONT long reads were aligned back to the assembly using minimap2. The depth of repeat and non-repeat regions was then calculated using bedtools. 26.06 Mb repeat sequences had been identified, accounting for 15.15% of the *C. cunea* genome, including DNA transposons (5.90%), unclassified (5.00%), retroelements (3.73%), LTR elements (2.60%), LINEs (1.12%), and simple repeats (0.16%). Rolling-circles and SINEs accounted for a small proportion of repetitive elements (Table 3, Table S1). Upon investigating the sequencing depths, we found that the mean depth within the repeat regions was approximately 243.82x, and for the non-repeat regions, it was about 261.07x. The modestly lower depth within the repeat regions, as compared to the non-repeat regions, suggests that there is no obvious collapse in the assembly. These repeat-sequences on all scaffolds were masked before genome annotation.

**Table 3:** Statistics of repetitive elements in the *C. cunea* genome.

Repeat type	Length occupied (bp)	Proportion in Genome
DNA transposon	10,149,350	5.90%
SINE	28,122	0.02%
LINE	1,925,055	1.12%
LTR	4,463,758	2.60%
Rolling-circles	197,149	0.11%
Satellite	432,465	0.25%
Simple repeat	275,891	0.16%
Unknown	8,591,132	5.00%
Total	26,062,922	15.15%

### Protein-coding genes (PCGs) prediction and other annotation of the genome

Protein-coding genes prediction was performed using three strategies: transcriptome-based prediction, homology-based prediction and ab initio prediction^26^. For transcriptome-based prediction, RNA-sequencing data derived from three larvae and adults were assembled separately using Hisat2 v2.2.1^27^ with default parameters, and then merged the three sets of results. The candidate coding region was identified by PASA v2.4.1^28^ with both gmap and blat aligners. Homology-based prediction was used Gemoma v1.7^29^ and Exonerate v2.4.0^30^ with default parameters and reference genomes of the model insect *Drosophila melanogaster* and six hymenopteran species: *Athalia rosae*, *Apis mellifera*, *N. vitripennis*, *Orussus abietinus*, *Venturia canescens*, and *Ceratosolen solmsi*. For ab initio prediction, Augustus v3.3.3^31^, SNAP v 2006-07-28^32^, and GeneMark-ES v4.57^33^, were trained by the high identity gene set and used to predict genes. All ab initio prediction software was executed with default parameters according to the manuals. Finally, all annotation results from the three strategies were integrated using EVidenceModeler (EVM) v1.1.1^34^. 12,258 protein-coding genes, 10,547 3′ -UTRs, and 10,671 5′ -UTRs were identified in this genome, the completeness of the annotation was evaluated by the BUSCO to be 98.70%, including 1,318 single-copy genes and 32 duplicated genes (Table S2). 11,277 genes (92.00%) encode proteins with at least one known domain in the InterPro database^35^.

For noncoding RNA (ncRNA) annotation, Infernal 1.1.2^36^ and tRNAScan v1.3^37^ were executed for rRNA and tRNA prediction, respectively. Other noncoding RNAs were detected by alignment against the Rfam database^38^. 431 noncoding RNAs (ncRNAs) were predicted, including 84 micro-RNAs (miRNAs), 97 ribosomal RNAs (rRNAs), 29 small nuclear RNAs (snRNAs), 16 small nucleolar RNAs (snoRNAs), and 205 transfer RNAs (tRNAs) (Table S3).

**Fig. 3:**
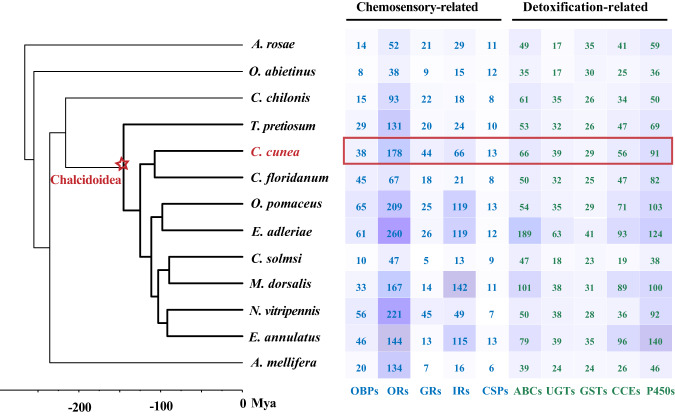
Counts for genes involved in chemosensory and detoxification in 13 representative hymenopterans. The red branches of phylogenetic tree indicate Chalcidoidea lineages. OBPs: odorant binding proteins; ORs: odorant receptors; GRs: gustatory receptors; IRs: ionotropic receptors; CSPs: chemosensory proteins; ABCs: ATP-binding cassette transporters; UGTs: UDP-glucosyltransferases; GSTs: gluthatione-S-transferases; CCEs: carboxylesterases; P450s: cytochrome P450.

We selected 12 other representative hymenopterans (Table S3), and all genes in 13 species genomes were annotated based on the databases of PANTHER (http://www.pantherdb.org/), CDD (http://www.ncbi.nlm.nih.gov/Structure/cdd/cdd.shtml), SUPERFAMILY (http://supfam.org/), and Pfam database (http://pfam.xfam.org/) by Interproscan. For gustatory receptors (GRs), ionotropic receptors (IRs), odorant receptors (ORs), odorant binding proteins (OBPs), chemosensory proteins (CSPs), UDP-glucosyltransferases (UGTs), gluthatione-S-transferases (GSTs), cytochrome P450 (P450s), carboxylesterases (CCEs), and ATP-binding cassette transporters (ABCs) identification, the domain of these genes (Table S7) were used to search for them. 17 GRs, 24 IRs, 154 ORs, 10 OBPs and 11 CSPs were identified in *C. cunea* (Fig. 3).

### Chromosome synteny

To identify collinear gene blocks between *C. cunea* and *N. vitripennis*, all protein coding genes were screened on genome-scale orthologue alignment (by blastp) using MCScanX^39^, and the syntenic genes of the high-quality blocks were visualized in Tbtools v0.58^40^. Syntenic relationships presented that 13,809 genes were linked between *C. cunea* and *N. vitripennis* (Fig. 2b). This demonstrated that the genome of *C. cunea* is highly homologous with that of *N. vitripennis*. Strong collinearity between these two species revealed conserved chromosomal characteristics.

### Gene family evolution and phylogenetic relationships

**Fig. 4:**
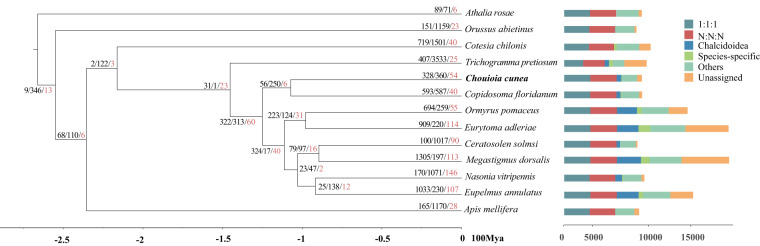
The maximum likelihood phylogenetic tree based on 2,986 concatenated single-copy orthologous genes from 13 hymenopterans. The bootstrap value of all nodes is supported at 100/100, and gene counts different types of orthologous groups. The expansion, contraction and rapid evolution numbers (red) of orthologous groups (OGs) are shown on the nodes and tips. “1:1:1” indicates universal single-copy genes present in all species; “N: N: N” indicates multicopy genes, although the absence in a single genome is tolerated; “Chalcidoidea” means common unique genes in species from Chalcidoidea. “Species-specific” represents species-specific genes in the genome; “Unassigned” indicates genes which cannot be assigned into any gene families (orthogroups); “Others” means the remaining genes.

The protein sequences with the longest transcript were kept to represent the coding region extracted from 13 species hymenopteran genomes including *A. rosae* (GCA_000344095.2), *O. abietinus* (GCF_000612105.2), *Eupelmus annulatus* (GCA_900480025.1), *N. vitripennis* (GCF_009193385.2), *Megastigmus dorsalis* (GCA_900490025.1), *C. solmsi* (GCA_000503995.1), *Ormyrus pomaceus* (GCA_900474385.1), *Eurytoma adleriae* (GCA_900480045.1), *C. cunea* (This study), *Copidosoma floridanum* (GCA_000648655.2), *Trichogramma pretiosum* (GCA_000599845.2), *Cotesia chilonis* (InsectBase ID: IBG_00206), *A. mellifera* (GCF_003254395.2). OrthoFinder v2.5.2^41^ was used to infer orthogroups (OGs; gene families), where 192,399 (89.5%) genes were assigned to 18,924 orthogroups. Among them, 4,606 orthogroups were identified as species specific, while 3,496 orthogroups were found in all species (Fig. 4). A total of 314 genes belonging to 54 families were identified as specific to *C. cunea*. Compared with other chalcidoids, *C. cunea* has fewer unique paralogs and annotated genes (Fig. 4). 2,986 single-copy genes were selected for phylogenetic tree construction. Protein sequences of the identified single-copy genes were aligned by MAFFT v7^42^, followed by Aliscore v02.2 and Alicut v2.31^43^ to remove the sites with weak phylogenetic signals. The best model (Q. plant + F + R5) using iqtree2 v2.1.3^44^ Model Finder Plus option, and iqtree2 (-m MFP -bb 1000) was used to construct the phylogenetic tree. Four calibration times based on the previous study^45^ and timetree database (timetree.org) were chosen for the divergence time estimation, Hymenoptera: 221–283 million years ago (mya), Chalcidoidea: 80–285 mya, *N. vitripennis* + *Eupelmus annulatus*: 44–112 mya, *Megastigmus dorsalis* + *C. solmsi*: 33–101 mya. Chalcidoidea is a monophyletic group as expected, and the topology shows the most consistency with previous studies^46,47^, and the origin of Chalcidoidea occurred during the Jurassic period (145–216 mya).

### Contraction and expansion of gene families

**Fig. 5:**
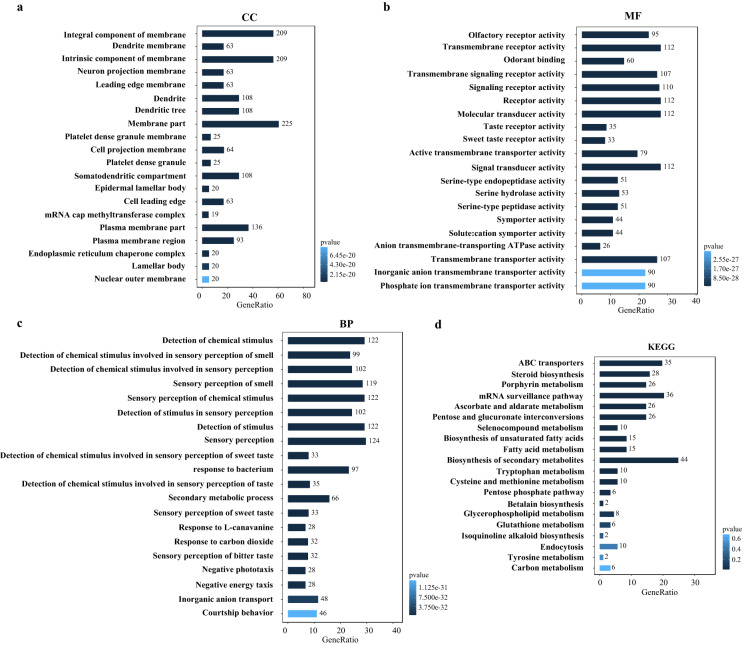
Enrichment analyses of expanded gene families of *C. cunea*. (**a**) GO enrichment of cellular component. The vertical axis represents the GO items, and the horizontal axis represents the gene ratio. The dot size indicates the number of genes, and the colour of the point corresponds to different adjusted p-value ranges. (**b**) GO enrichment of molecular function. (**c**) GO enrichment of biological process. (**d**) KEGG enrichment map of expanded gene families. The vertical axis represents the path name, and the horizontal axis represents the gene ratio. The dot size indicates the number of genes, and the colour of the point corresponds to different p value ranges.

OGs evolution (expansion and contraction) was calculated to birth and death rates using CAFE v5.0^48^ with the lambda parameter based on the tree above. In *C. cunea*, 11,691 (95.40%) genes were clustered into 9,204 gene families, of which 328 gene families were expanded, 360 gene families were contracted, and 54 (37 expansions and 17 contractions) gene families experienced rapid evolution. These gene functions were illustrated by comprehensive databases, such as Gene Ontology (GO) and Kyoto Encyclopedia of Genes and Genomes (KEGG). Functional annotation was performed using emapper v2.1.3 with hmmer v3.3.2 sequence searches strategy and assigning to eggNOG orthology data of Insecta^49–51^. The significantly expanded families were involved in various molecular functions and biological processes (Fig. 5, Table S5), including trypsin-like serine protease, odorant receptor, gustatory receptor, sugar transporter, cytochrome P450, ABC transporter and alpha/beta hydrolase encoding genes. The families with significant contractions mainly include the DDE superfamily endonuclease, putative peptidase, and hAT family C-terminal dimerisation region (Table S6). All gene enrichments of the KEGG pathway and GO terms were executed by R package clusterProfiler v4.1.1 (Fig. 5)^52^.

## Data Records

All raw data of the whole genome have been deposited into the National Center for Biotechnology Information (NCBI) under BioProject accession number PRJNA881787.

The genomic sequencing data were deposited in the SRA at NCBI SRR21622056^53^ and SRR21655425^54^.

The transcriptomic sequencing data were deposited in the SRA at NCBI SRR21620783, SRR21621373 and SRR21621151^55–57^.

The Hi-C sequencing data were deposited in the SRA at NCBI SRR21620083^58^.

The final genome assembly was deposited in GenBank at NCBI JAOPFQ000000000^59^.

Results from the genome assembly and annotation are available in figshare^60^.

## Technical Validation

### Quality evaluation of genome assembly

We assessed the quality of *C. cunea* genome assembly in the three aspects. Firstly, the Core Eukaryotic Genes Mapping Approach (CEGMA) defined 458 core eukaryotic genes, and 248 of them were the most highly-conserved core genes, which could be used to assess the completeness of the genome or annotations. We identified that 245 (98.79%) core genes have homologous genes in the highly-conserved core gene sets. Secondly, the completeness of protein-coding gene predictions was evaluated by the BUSCO (in the insects_odb10 database) completeness to be 98.70% (96.4% single-copied genes and 2.3% duplicated genes), 0.1% fragmented, and 1.2% missing. Thirdly, sequencing data were mapped to the genome to verify the accuracy, yielding mapping rates of 99.90% for MGI, and 98.85% for ONT data.

## Supplementary information

SUPPLEMENTARY INFORMATION

## Data Availability

The specific scripts have been deposited in figshare. All data analyses were performed according to the manual and protocols of the published bioinformatic tools. The version and parameters of software have been described in Methods section.
