# Supplementary material for: Chromosome-level genome assembly of Chouioia cunea Yang, the parasitic wasp of the fall webworm
Source: Sci Data. 2023 Jul 26;10:485. doi: 10.1038/s41597-023-02388-5 (PMC10372138; doi:10.1038/s41597-023-02388-5)
Supplement: Supplementary file 1 — SUPPLEMENTARY INFORMATION [file 41597_2023_2388_MOESM1_ESM.docx]

**Supplementary information of Chromosome-level assembly of *Chouioia cunea* genome**

| Table S1. Repeat annotation in the *Chouioia cunea* genome. | Page2 |
| --- | --- |
| Table S2 BUSCOs for *C. cunea* genome at the protein level to insect. | Page3 |
| Table S3. Annotations of non-coding RNAs in the *C. cunea* genome. | Page3 |
| Table S4. Species taxonomic information and accession code of 13 species used to gene family evolution and phylogenetic relationships analyses. | Page9 |
| Table S5. The list of significant expanded families in *C. cunea* genome. | Page9 |
| Table S6. The list of significant contracted families in *C. cunea* genome. | Page11 |
| Table S7. Domain used for searching interested gene families. | Page11 |

**Table S1. Repeat annotation in the *Chouioia cunea* genome.**

| **Class** | | Count | bpMasked | %masked |
| --- | --- | --- | --- | --- |
| **Retroelements** | | 28430 | 6416935bp | 3.73% |
|  | SINEs: | 332 | 28122bp | 0.02% |
|  | Penelope | 124 | 5197 bp | 0.00% |
|  | LINEs: | 12135 | 1925055 bp | 1.12% |
|  | CRE/SLACS | 2 | 236 bp | 0.00% |
|  | L2/CR1/Rex | 3326 | 161807 bp | 0.09% |
|  | R1/LOA/Jockey | 5978 | 704910 bp | 0.41% |
|  | R2/R4/NeSL | 11 | 444 bp | 0.00% |
|  | RTE/Bov-B | 32 | 4193 bp | 0.00% |
|  | L1/CIN4 | 148 | 8379 bp | 0.00% |
|  | LTR elements: | 15963 | 4463758 bp | 2.60% |
|  | BEL/Pao | 2998 | 294404 bp | 0.17% |
|  | Ty1/Copia | 278 | 19958 bp | 0.01% |
|  | Gypsy/DIRS1 | 8427 | 3869592 bp | 2.25% |
|  | Retroviral | 2777 | 155824 bp | 0.09% |
| **DNA transposons** | | 59598 | 10149350 bp | 5.90% |
|  | hobo-Activator | 7713 | 438580 bp | 0.26% |
|  | Tc1-IS630-Pogo | 5204 | 1255427 bp | 0.73% |
|  | En-Spm | 0 | 0 bp | 0.00% |
|  | MuDR-IS905 | 0 | 0 bp | 0.00% |
|  | PiggyBac | 1052 | 571537 bp | 0.33% |
|  | Tourist/Harbinger | 1197 | 74891 bp | 0.04% |
|  | Other (Mirage, P-element,Transib) | 200 | 9161 bp | 0.01% |
| **Rolling-circles** | | 1387 | 197149 bp | 0.11% |
| **Unclassified** | | 35017 | 8591132 bp | 5.00% |
| **Total interspersed repeats** | |  | 25157417 bp | 14.63% |
| **Small RNA** | | 0 | 0bp | 0.00% |
| **Satellites** | | 7038 | 432465 bp | 0.25% |
| **Simple repeats** | | 5314 | 275891 bp | 0.16% |
| **Low complexity** | | 0 | 0bp | 0.00% |

**Table S2 BUSCOs for *C. cunea* genome at the protein level to insect**

|  | **BUSCOs** | **Number** | **Percent** |
| --- | --- | --- | --- |
| **BUSCOs for assembly** | Complete BUSCOs | 1349 | 98.70% |
|  | Complete and single-copy BUSCOs (S) | 1323 | 96.80% |
|  | Complete and duplicated BUSCOs (D) | 26 | 1.90% |
|  | Fragmented BUSCOs (F) | 1 | 0.10% |
|  | Missing BUSCOs (M) | 17 | 1.20% |
| **BUSCOs for annotation** | Complete BUSCOs | 1350 | 98.70% |
|  | Complete and single-copy BUSCOs (S) | 1318 | 96.40% |
|  | Complete and duplicated BUSCOs (D) | 32 | 3.20% |
|  | Fragmented BUSCOs (F) | 2 | 0.10% |
|  | Missing BUSCOs (M) | 15 | 1.20% |

**Table S3. Annotations of non-coding RNAs in the *C. cunea* genome**

| **Class** | **Copy Number** | **Description** |
| --- | --- | --- |
| **rRNA** | 97 |  |
| Ccun_rRNA_17 | 1 | rRNA |
| Ccun_rRNA_18 | 1 | rRNA |
| Ccun_rRNA_19 | 1 | rRNA |
| Ccun_rRNA_20 | 1 | rRNA |
| Ccun_rRNA_21 | 1 | rRNA |
| Ccun_rRNA_22 | 1 | rRNA |
| Ccun_rRNA_23 | 1 | rRNA |
| Ccun_rRNA_24 | 1 | rRNA |
| Ccun_rRNA_25 | 1 | rRNA |
| Ccun_rRNA_26 | 1 | rRNA |
| Ccun_rRNA_27 | 1 | rRNA |
| Ccun_rRNA_28 | 1 | rRNA |
| Ccun_rRNA_29 | 1 | rRNA |
| Ccun_rRNA_30 | 1 | rRNA |
| Ccun_rRNA_31 | 1 | rRNA |
| Ccun_rRNA_32 | 1 | rRNA |
| Ccun_rRNA_33 | 1 | rRNA |
| Ccun_rRNA_34 | 1 | rRNA |
| Ccun_rRNA_35 | 1 | rRNA |
| Ccun_rRNA_36 | 1 | rRNA |
| Ccun_rRNA_37 | 1 | rRNA |
| Ccun_rRNA_38 | 1 | rRNA |
| Ccun_rRNA_39 | 1 | rRNA |
| Ccun_rRNA_40 | 1 | rRNA |
| Ccun_rRNA_41 | 1 | rRNA |
| Ccun_rRNA_42 | 1 | rRNA |
| Ccun_rRNA_43 | 1 | rRNA |
| Ccun_rRNA_44 | 1 | rRNA |
| Ccun_rRNA_45 | 1 | rRNA |
| Ccun_rRNA_46 | 1 | rRNA |
| Ccun_rRNA_47 | 1 | rRNA |
| Ccun_rRNA_48 | 1 | rRNA |
| Ccun_rRNA_49 | 1 | rRNA |
| Ccun_rRNA_50 | 1 | rRNA |
| Ccun_rRNA_51 | 1 | rRNA |
| Ccun_rRNA_52 | 1 | rRNA |
| Ccun_rRNA_53 | 1 | rRNA |
| Ccun_rRNA_54 | 1 | rRNA |
| Ccun_rRNA_55 | 1 | rRNA |
| Ccun_rRNA_56 | 1 | rRNA |
| Ccun_rRNA_57 | 1 | rRNA |
| Ccun_rRNA_58 | 1 | rRNA |
| Ccun_rRNA_59 | 1 | rRNA |
| Ccun_rRNA_60 | 1 | rRNA |
| Ccun_rRNA_61 | 1 | rRNA |
| Ccun_rRNA_62 | 1 | rRNA |
| Ccun_rRNA_63 | 1 | rRNA |
| Ccun_rRNA_64 | 1 | rRNA |
| Ccun_rRNA_65 | 1 | rRNA |
| Ccun_rRNA_66 | 1 | rRNA |
| Ccun_rRNA_67 | 1 | rRNA |
| Ccun_rRNA_68 | 1 | rRNA |
| Ccun_rRNA_69 | 1 | rRNA |
| Ccun_rRNA_70 | 1 | rRNA |
| Ccun_rRNA_71 | 1 | rRNA |
| Ccun_rRNA_72 | 1 | rRNA |
| Ccun_rRNA_73 | 1 | rRNA |
| Ccun_rRNA_74 | 1 | rRNA |
| Ccun_rRNA_75 | 1 | rRNA |
| Ccun_rRNA_76 | 1 | rRNA |
| Ccun_rRNA_77 | 1 | rRNA |
| Ccun_rRNA_78 | 1 | rRNA |
| Ccun_rRNA_79 | 1 | rRNA |
| Ccun_rRNA_80 | 1 | rRNA |
| Ccun_rRNA_81 | 1 | rRNA |
| Ccun_rRNA_82 | 1 | rRNA |
| Ccun_rRNA_83 | 1 | rRNA |
| Ccun_rRNA_84 | 1 | rRNA |
| Ccun_rRNA_85 | 1 | rRNA |
| Ccun_rRNA_86 | 1 | rRNA |
| Ccun_rRNA_87 | 1 | rRNA |
| Ccun_rRNA_88 | 1 | rRNA |
| Ccun_rRNA_89 | 1 | rRNA |
| Ccun_rRNA_90 | 1 | rRNA |
| Ccun_rRNA_91 | 1 | rRNA |
| Ccun_rRNA_92 | 1 | rRNA |
| Ccun_rRNA_93 | 1 | rRNA |
| Ccun_rRNA_94 | 1 | rRNA |
| Ccun_rRNA_95 | 1 | rRNA |
| Ccun_rRNA_96 | 1 | rRNA |
| Ccun_rRNA_97 | 1 | rRNA |
| Ccun_rRNA_98 | 1 | rRNA |
| Ccun_rRNA_99 | 1 | rRNA |
| Ccun_rRNA_100 | 1 | rRNA |
| Ccun_rRNA_101 | 1 | rRNA |
| Ccun_rRNA_102 | 1 | rRNA |
| Ccun_rRNA_103 | 1 | rRNA |
| Ccun_rRNA_104 | 1 | rRNA |
| Ccun_rRNA_105 | 1 | rRNA |
| Ccun_rRNA_106 | 1 | rRNA |
| Ccun_rRNA_107 | 1 | rRNA |
| Ccun_rRNA_108 | 1 | rRNA |
| Ccun_rRNA_109 | 1 | rRNA |
| Ccun_rRNA_110 | 1 | rRNA |
| Ccun_rRNA_111 | 1 | rRNA |
| Ccun_rRNA_112 | 1 | rRNA |
| Ccun_rRNA_113 | 1 | rRNA |
| **miRNA** | 84 |  |
| Ccun-mir-965 | 1 | miRNA |
| Ccun-mir-133 | 1 | miRNA |
| Ccun-mir-1 | 1 | miRNA |
| Ccun-Mir-307 | 1 | miRNA |
| Ccun-mir-283 | 1 | miRNA |
| Ccun-mir-3477 | 1 | miRNA |
| Ccun-mir-12 | 1 | miRNA |
| Ccun-mir-927a | 1 | miRNA |
| Ccun-mir-956 | 1 | miRNA |
| Ccun-mir-6012 | 1 | miRNA |
| Ccun-mir-11 | 1 | miRNA |
| Ccun-mir-34 | 1 | miRNA |
| Ccun-mir-277 | 1 | miRNA |
| Ccun-mir-317 | 1 | miRNA |
| Ccun-mir-iab-8 | 1 | miRNA |
| Ccun-mir-iab-4 | 1 | miRNA |
| Ccun-mir-10 | 1 | miRNA |
| Ccun-mir-993 | 1 | miRNA |
| Ccun-mir-2796 | 1 | miRNA |
| Ccun-mir-279a | 1 | miRNA |
| Ccun-mir-279c | 1 | miRNA |
| Ccun-mir-275 | 1 | miRNA |
| Ccun-mir-305 | 1 | miRNA |
| Ccun-mir-124 | 1 | miRNA |
| Ccun-mir-276 | 1 | miRNA |
| Ccun-mir-184 | 1 | miRNA |
| Ccun-mir-318 | 1 | miRNA |
| Ccun-mir-4 | 1 | miRNA |
| Ccun-mir-279b | 1 | miRNA |
| Ccun-mir-3791 | 1 | miRNA |
| Ccun-mir-971 | 1 | miRNA |
| Ccun-mir-29b | 1 | miRNA |
| Ccun-mir-6039 | 1 | miRNA |
| Ccun-Mir-14 | 1 | miRNA |
| Ccun-Mir-100 | 1 | miRNA |
| Ccun-let-7 | 1 | miRNA |
| Ccun-mir-980 | 1 | miRNA |
| Ccun-mir-219 | 1 | miRNA |
| Ccun-mir-71 | 1 | miRNA |
| Ccun-mir-2a-1 | 1 | miRNA |
| Ccun-mir-13a | 1 | miRNA |
| Ccun-mir-13b | 1 | miRNA |
| Ccun-mir-2a-2 | 1 | miRNA |
| Ccun-mir-2a-3 | 1 | miRNA |
| Ccun-mir-6037 | 1 | miRNA |
| Ccun-bantam | 1 | miRNA |
| Ccun-mir-9a | 1 | miRNA |
| Ccun-mir-315 | 1 | miRNA |
| Ccun-mir-210 | 1 | miRNA |
| Ccun-mir-6038 | 1 | miRNA |
| Ccun-mir-3715a | 1 | miRNA |
| Ccun-mir-3715b | 1 | miRNA |
| Ccun-mir-96b | 1 | miRNA |
| Ccun-mir-279d | 1 | miRNA |
| Ccun-mir-263 | 1 | miRNA |
| Ccun-mir-2765 | 1 | miRNA |
| Ccun-Mir-1175 | 1 | miRNA |
| Ccun-mir-750 | 1 | miRNA |
| Ccun-mir-92b | 1 | miRNA |
| Ccun-mir-92a | 1 | miRNA |
| Ccun-mir-33 | 1 | miRNA |
| Ccun-mir-193 | 1 | miRNA |
| Ccun-mir-2788 | 1 | miRNA |
| Ccun-mir-375 | 1 | miRNA |
| Ccun-mir-8 | 1 | miRNA |
| Ccun-mir-927b | 1 | miRNA |
| Ccun-mir-31 | 1 | miRNA |
| Ccun-mir-190 | 1 | miRNA |
| Ccun-mir-92c | 1 | miRNA |
| Ccun-mir-7 | 1 | miRNA |
| Ccun-mir-137 | 1 | miRNA |
| Ccun-mir-929 | 1 | miRNA |
| Ccun-mir-6001 | 1 | miRNA |
| Ccun-Mir-252a | 1 | miRNA |
| Ccun-mir-252b | 1 | miRNA |
| Ccun-mir-6057 | 1 | miRNA |
| Ccun-mir-281 | 1 | miRNA |
| Ccun-mir-3783 | 1 | miRNA |
| Ccun-mir-1000 | 1 | miRNA |
| Ccun-mir-932 | 1 | miRNA |
| Ccun-mir-3049 | 1 | miRNA |
| Ccun-mir-981 | 1 | miRNA |
| Ccun-mir-989 | 1 | miRNA |
| Ccun-mir-2944 | 1 | miRNA |
| **snRNA** | 29 |  |
| Ccun_snRNA_114 | 1 | snRNA |
| Ccun_snRNA_115 | 1 | snRNA |
| Ccun_snRNA_116 | 1 | snRNA |
| Ccun_snRNA_117 | 1 | snRNA |
| Ccun_snRNA_118 | 1 | snRNA |
| Ccun_snRNA_119 | 1 | snRNA |
| Ccun_snRNA_120 | 1 | snRNA |
| Ccun_snRNA_121 | 1 | snRNA |
| Ccun_snRNA_122 | 1 | snRNA |
| Ccun_snRNA_123 | 1 | snRNA |
| Ccun_snRNA_124 | 1 | snRNA |
| Ccun_snRNA_125 | 1 | snRNA |
| Ccun_snRNA_126 | 1 | snRNA |
| Ccun_snRNA_127 | 1 | snRNA |
| Ccun_snRNA_128 | 1 | snRNA |
| Ccun_snRNA_129 | 1 | snRNA |
| Ccun_snRNA_130 | 1 | snRNA |
| Ccun_snRNA_131 | 1 | snRNA |
| Ccun_snRNA_132 | 1 | snRNA |
| Ccun_snRNA_133 | 1 | snRNA |
| Ccun_snRNA_134 | 1 | snRNA |
| Ccun_snRNA_135 | 1 | snRNA |
| Ccun_snRNA_136 | 1 | snRNA |
| Ccun_snRNA_137 | 1 | snRNA |
| Ccun_snRNA_138 | 1 | snRNA |
| Ccun_snRNA_139 | 1 | snRNA |
| Ccun_snRNA_140 | 1 | snRNA |
| Ccun_snRNA_141 | 1 | snRNA |
| Ccun_snRNA_142 | 1 | snRNA |
| **snoRNA** | 16 |  |
| Ccun_snoRNA_1 | 1 | snoRNA |
| Ccun_snoRNA_2 | 1 | snoRNA |
| Ccun_snoRNA_3 | 1 | snoRNA |
| Ccun_snoRNA_4 | 1 | snoRNA |
| Ccun_snoRNA_5 | 1 | snoRNA |
| Ccun_snoRNA_6 | 1 | snoRNA |
| Ccun_snoRNA_7 | 1 | snoRNA |
| Ccun_snoRNA_8 | 1 | snoRNA |
| Ccun_snoRNA_9 | 1 | snoRNA |
| Ccun_snoRNA_10 | 1 | snoRNA |
| Ccun_snoRNA_11 | 1 | snoRNA |
| Ccun_snoRNA_12 | 1 | snoRNA |
| Ccun_snoRNA_13 | 1 | snoRNA |
| Ccun_snoRNA_14 | 1 | snoRNA |
| Ccun_snoRNA_15 | 1 | snoRNA |
| Ccun_snoRNA_16 | 1 | snoRNA |
| **tRNA** | 205 | tRNA |
| tRNAs with introns | 17 | tRNA |
| Leu-CAA | 3 | tRNA |
| Asn-ATT | 1 | tRNA |
| Ile-TAT | 2 | tRNA |
| Tyr-ATA | 3 | tRNA |
| Tyr-GTA | 8 | tRNA |
| Isotype / Anticodon Counts: | 188 | tRNA |
| tRNA-Ala | 15 | tRNA |
| tRNA-Arg | 11 | tRNA |
| tRNA-Asp | 9 | tRNA |
| tRNA-Asn | 8 | tRNA |
| tRNA-Cys | 5 | tRNA |
| tRNA-Gln | 8 | tRNA |
| tRNA-Glu | 15 | tRNA |
| tRNA-Gly | 15 | tRNA |
| tRNA-His | 6 | tRNA |
| tRNA-Ile | 9 | tRNA |
| tRNA-Leu | 16 | tRNA |
| tRNA-Lys | 15 | tRNA |
| tRNA-Met | 4 | tRNA |
| tRNA-Phe | 5 | tRNA |
| tRNA-Pro | 11 | tRNA |
| tRNA-Ser | 12 | tRNA |
| tRNA-Thr | 8 | tRNA |
| tRNA-Trp | 4 | tRNA |
| tRNA-Tyr | 11 | tRNA |
| tRNA-Val | 12 | tRNA |
| **Total** | 431 |  |

**Table S4. Species taxonomic information and accession code of 13 species used to gene family evolution and phylogenetic relationships analyses.**

| **Order** | **Subfamily** | **Family** | **Species** | **Accession number** | **Source** |
| --- | --- | --- | --- | --- | --- |
| Hymenoptera | Chalcidoidea | Tentnredinidae | *Athalia rosae* | GCA_000344095.2 | NCBI |
| Hymenoptera | Chalcidoidea | Orussidae | *Orussus abietinus* | GCF_000612105.2 | NCBI |
| Hymenoptera | Chalcidoidea | Eupelmidae | *Eupelmus annulatus* | GCA_900480025.1 | NCBI |
| Hymenoptera | Chalcidoidea | Pteromalidae | *Nasonia vitripennis* | GCF_009193385.2 | NCBI |
| Hymenoptera | Chalcidoidea | Megastigmidae | *Megastigmus dorsalis* | GCA_900490025.1 | NCBI |
| Hymenoptera | Chalcidoidea | Agaonidae | *Ceratosolen solmsi* | GCA_000503995.1 | NCBI |
| Hymenoptera | Chalcidoidea | Ormyridae | *Ormyrus pomaceus* | GCA_900474385.1 | NCBI |
| Hymenoptera | Chalcidoidea | Eurytomidae | *Eurytoma adleriae* | GCA_900480045.1 | NCBI |
| Hymenoptera | Chalcidoidea | Eulophidae | *Chouioia cunea* | - | This study |
| Hymenoptera | Chalcidoidea | Encyrtidae | *Copidosoma floridanum* | GCA_000648655.2 | NCBI |
| Hymenoptera | Chalcidoidea | Trichogrammatidae | *Trichogramma brassicae* | GCA_902806795.1 | NCBI |
| Hymenoptera | Chalcidoidea | Braconidae | *Cotesia chilonis* | InsectBase ID: IBG_00206 | InsectBase |
| Hymenoptera | Chalcidoidea | Apoidae | *Apis mellifera* | GCF_003254395.2 | NCBI |

**Table S5. The list of significant expanded families in *C. cunea* genome**

| **Desc** | **seed_ortholog** | **Description** | **Preferred_name** |
| --- | --- | --- | --- |
| OG0000009 | 7425.NV14768-PA | Trypsin-like serine protease | - |
| OG0000014 | 7425.NV15591-PA | Cytochrome P450 | - |
| OG0000015 | 7460.GB50195-PA | ABC transporter transmembrane region | ABCC4 |
| OG0000018 | 7425.NV18591-PA | UDP-glucoronosyl and UDP-glucosyl transferase | UGT2A1 |
| OG0000024 | 7425.NV22370-PA | 7tm Odorant receptor | GPROR31 |
| OG0000029 | 7425.NV13723-PA | Sugar (and other) transporter | - |
| OG0000056 | 7425.NV10469-PA | alpha/beta hydrolase fold | - |
| OG0000063 | 7425.NV16839-PA | 7tm Odorant receptor | Or63a |
| OG0000069 | 7425.NV10373-PA | Trypsin-like serine protease | KLK7 |
| OG0000072 | 7425.NV17609-PA | Sugar (and other) transporter | - |
| OG0000092 | 103372.F4W867 | Gustatory receptor which mediates acceptance or avoidance behavior, depending on its substrates | Gr28a |
| OG0000095 | 7425.NV18647-PA | Integrase core domain | - |
| OG0000097 | 7425.NV23334-PA | 7tm Odorant receptor | - |
| OG0000099 | 7425.NV15989-PA | Endothelin-converting enzyme | Nep2 |
| OG0000102 | 7425.NV15710-PA | Belongs to the AB hydrolase superfamily. Lipase family | - |
| OG0000109 | 7425.NV24097-PA | calcium ion binding | - |
| OG0000139 | 7425.NV20649-PA | ionotropic glutamate receptor activity | - |
| OG0000142 | 7425.NV14634-PA | Venom acid phosphatase | - |
| OG0000148 | 7425.NV21444-PA | Aminotransferase class I and II | CCBL2 |
| OG0000158 | 7425.NV16696-PA | Common central domain of tyrosinase | PPO1 |
| OG0000179 | 7425.NV13310-PA | ZnF_C4 abd HLH domain containing kinases domain | - |
| OG0000182 | 7425.NV18554-PA | L-lysine 6-monooxygenase (NADPH-requiring) | - |
| OG0000194 | 7425.NV12990-PA | ABC-2 family transporter protein | - |
| OG0000219 | 7425.NV22676-PA | - | - |
| OG0000244 | 7425.NV16712-PA | Domain of unknown function (DUF4371) | - |
| OG0000254 | 7425.NV15499-PA | Speckle-type POZ protein | SPOPL |
| OG0000337 | 7425.NV18726-PA | Poly(A) polymerase central domain | hrg |
| OG0000364 | 7425.NV14126-PA | Major royal jelly protein | - |
| OG0000370 | 7460.GB46579-PA | Catalyzes the rate-limiting step of the oxidative pentose-phosphate pathway, which represents a route for the dissimilation of carbohydrates besides glycolysis | G6PD |
| OG0000393 | 7425.NV15032-PA | Belongs to the class I-like SAM-binding methyltransferase superfamily. mRNA cap 0 methyltransferase family | RNMT |
| OG0000449 | 7425.NV19087-PA | Odorant receptor | - |
| OG0000649 | 7425.NV10269-PA | Possible membrane-associated motif in LPS-induced tumor necrosis factor alpha factor (LITAF), also known as PIG7, and other animal proteins. | - |
| OG0000651 | 103372.F4W5A5 | Fatty acid desaturase | SCD |
| OG0000910 | 121225.PHUM269500-PA | DNA binding | - |
| OG0001363 | 7425.NV14732-PA | Sugar (and other) transporter | - |
| OG0001403 | 7425.NV13487-PA | Lipocalin-like domain | - |
| OG0002651 | 7425.NV11844-PA | N-acetyltransferase activity | GLYATL3 |

**Table S6. The list of significant contracted families in *C. cunea* genome**

| **Desc** | **seed_ortholog** | **Description** | **Preferred_name** |
| --- | --- | --- | --- |
| OG0000006 | 7370.XP_005192252.1 | Putative peptidase (DUF1758) | - |
| OG0000019 | 7425.NV23927-PA | - | - |
| OG0000021 | 7425.NV24909-PA | - | - |
| OG0000052 | 7425.NV26225-PA | DDE superfamily endonuclease | - |
| OG0000149 | 7425.NV10450-PA | Zn_pept | - |
| OG0000008 | 7425.NV22419-PA | DDE superfamily endonuclease | - |
| OG0000034 | 7425.NV24477-PA | DDE superfamily endonuclease | - |
| OG0000053 | 7425.NV18608-PA | Integrase core domain | - |
| OG0000115 | 69319.XP_008553664.1 | oxidation-reduction process | - |
| OG0000124 | 7070.TC010509-PA | protein heterodimerization activity | - |
| OG0000155 | 7425.NV21493-PA | MAP kinase kinase kinase activity | - |
| OG0000167 | 69319.XP_008558656.1 | Putative peptidase (DUF1758) | - |
| OG0000168 | 7029.ACYPI080370-PA | G-quadruplex DNA unwinding | - |
| OG0000284 | 7425.NV25056-PA | DDE superfamily endonuclease | - |
| OG0000336 | 7425.NV25054-PA | transcription regulator activity | - |
| OG0000425 | 6087.XP_004211325.1 | hAT family C-terminal dimerisation region | - |
| OG0000457 | 7425.NV24231-PA | Reverse transcriptase (RNA-dependent DNA polymerase) | - |

**Table S7. Domain used for searching interested gene families**

| **Gene family** | **Domains** |
| --- | --- |
| Gustatory receptors (GRs) | PTHR21143 PTHR21431 |
| Ionotropic receptors (IRs) | PTHR42642 PTHR18966 PF00060 |
| Odorant receptors (ORs) | PF02949 PTHR21137 |
| Odorant binding proteins (OBPs) | PF01395 |
| Chemosensory proteins (CSPs) | PTHR11257 SSF100910 |
| UDP-glucosyltransferases (UGTs) | PF00534 cd03784 PF03360 PF02366 SSF53756 PF00852 PF00201 |
| Gluthatione-S-transferases (GSTs) | PF00043 PF17171 PF13417 PF02798 PF14497 PF13410 |
| Cytochrome P450 (P450s) | PF00067 |
| Carboxylesterases (CCEs) | PTHR11559 PF00135 PF02230 |
| ATP-binding cassette transporters (ABCs) | PF00005 PF19055 PF01061 |
